# Investigation of a New Flux-Modulated Permanent Magnet Brushless Motor for EVs

**DOI:** 10.1155/2014/540797

**Published:** 2014-04-16

**Authors:** Ying Fan, Lingling Gu, Yong Luo, Xuedong Han, Ming Cheng

**Affiliations:** ^1^School of Electrical Engineering, Southeast University, Nanjing 210096, China; ^2^Jiangsu Key Laboratory of Smart Grid Technology and Equipment, Zhenjiang 212009, China; ^3^Department of New Energy, Jiangsu Electric Power Design Institute, Nanjing 2111100, China

## Abstract

This paper presents a flux-modulated direct drive (FMDD) motor. The key is to integrate the magnetic gear with the PM motor while removing the gear inner-rotor. Hence, the proposed FMDD motor can achieve the low-speed high-torque output and high-speed compact design requirements as well as high-torque density with a simple structure. The output power equation is analytically derived. By using finite element analysis (FEA), the static characteristics of the proposed motor are obtained. Based on these characteristics, the system mathematical model can be established. Hence, the evaluation of system performances is conducted by computer simulation using the Matlab/Simulink. A prototype is designed and built for experimentation. Experimental results are given to verify the theoretical analysis and simulation.

## 1. Introduction

To improve the fuel economy and reduce the emissions in the transportation areas, electric vehicles (EVs) and hybrid electric vehicles (HEVs) are the most viable solution [1]. As the key parts of EVs and HEVs, electric motors can be classified into two types. One is the concentrate motor driving [2]; the wheels are driven by motor through planetary gears and gearboxes indirectly.  However,  it causes the maintenance, transmission loss, and acoustic noise. The other is the in-wheel motor driving; especially the PM in-wheel motors have been paid much attention, which have the advantages of simple structure and high efficiency. There are two types of PM in-wheel motors; one is the outer-rotor topology without gear; another is the inner-rotor one in which a planetary gear is employed. The former can provide the low-speed operation directly, but it causes big size and heavy weight. On the other hand, the latter has the advantages of reduced size and weight, but the planetary gear has the defects such as lubrication, transmission loss, and acoustic noise [3].

**Figure 1:**
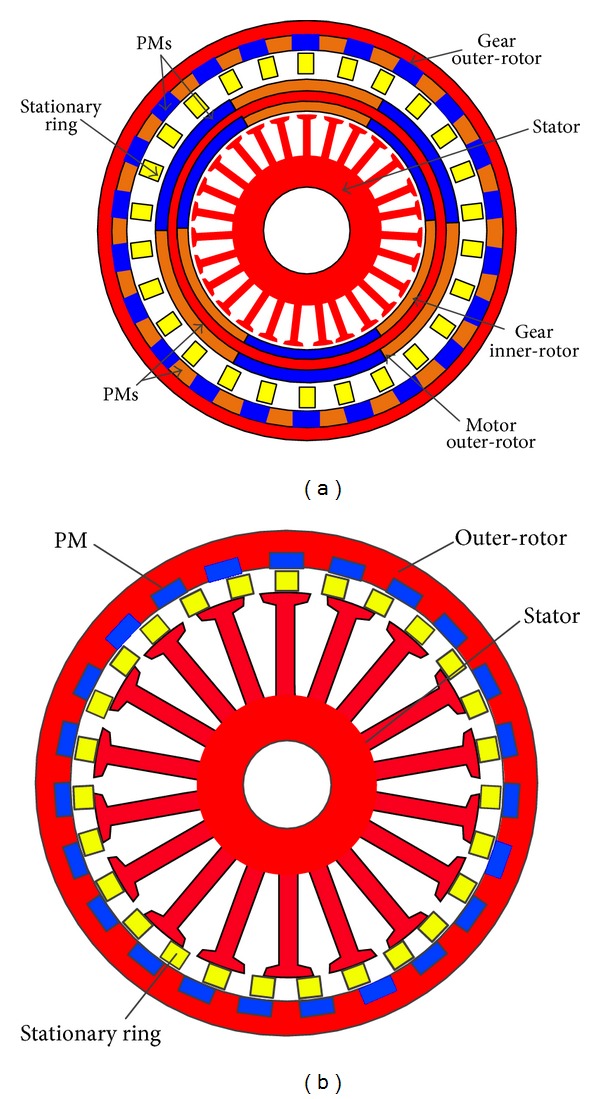
Configuration of magnetic-geared PM motor: (a) three air-gaps and (b) the proposed motor.

With the advent of magnetic gears, magnetic transmission systems have been developed quickly [4]. The magnetic gear has some distinct advantages when compared to mechanical gears: no mechanical fatigue; no lubrication; overload protected; no contact losses; no transmission contact acoustic noise; high efficiency (only a little core loss and bearing loss); and high torque per volume ratio (ten times the standard motors) [5]. When such a magnetic gear is coupled with a conventional PM motor, the overall torque density can be significantly improved. Based on this combination, a magnetic-geared PM motor is proposed to be used as a direct drive motor [6].  Figure 1(a) shows the configuration of this magnetic-geared PM motor with three air-gaps, which consists of four parts: the stator, modulation ring, inner rotor, and outer-rotor. The modulation ring is used to modulate the air-gap field space harmonics. The PMs are buried in the iron core of both rotors and magnetized in alien-polarity. However, the complicated structure causes manufacturing difficulty and instability. Furthermore, its power density is limited by its high flux leakage and iron loss. In [7], an improved topology with two air-gaps is proposed, in which the high-speed inner-rotor is omitted, so the structure is simple. But the outer-rotor is the same as the three air-gap topology. In [8], another fractional-slot flux-modulated PM motor with two air-gaps is developed. The rule for comparing the power density of electric motors is proposed and its cogging torque is very small. It also confirms that the magnetic-geared PM motor is a better choice than the conventional PM motor for low-speed drives. However, the same requirement of two or three air-gaps as that in [5–9] will lead to the same problems. Furthermore, experimental results are rarely given to verify the design, analysis, and control of magnetic-geared PM motors.

The major contribution of this paper is to propose and implement a flux-modulated direct drive (FMDD) motor, having a simple structure while incorporating the advantages of PM machines and magnetic gears. This paper will focus on the design, analysis, control, and experimental verification of the proposed motor. The motor configuration and operating principles will be described in Section 2. The design criteria of the motor are presented in Section 3.  Section 4 will be devoted to the electromagnetic analysis. The two-dimensional (2D) FEA is used to analyze the static performance of the motor. In Section 5, based on Matlab/Simulink, simulation of the whole drive system will be discussed. The implementation of test-bed and experimental results will also be given for verification. Finally, conclusion will be drawn in Section 6.

## 2. Motor Configurations and Operating Principle

### 2.1. Motor Topology

As shown in Figure 1(b), it is the proposed flux-modulated direct drive motor for low-speed drives. The coaxial magnetic gear is integrated into a PM machine; this gear is composed of three parts which are stator, outer-rotor, and stationary ring between them. It should be noted that the stationary ring can be separated into two parts: one is the flux modulation block which is made from laminated iron core, and it provides the path for magnetic field, and the remaining part is filled with epoxy which is nonferromagnetic. The high-speed rotating field of the inner armature windings is modulated into the low-speed rotating field of the PM outer-rotor. Hence, the outer-rotor can provide a high-torque output while operating at a low speed.

All the PMs are embedded in the outer-rotor along the circumferential periphery and magnetized in the same direction. PM flux flowing in the iron core is equivalent with that magnetized in the alien direction, thus creating a distributed magnetic field with the same number of pole pairs. Compared with the motor in Figure 1(a), the PM materials of the proposed motor can be saved, and the structure is simplified. Hence, cost can be reduced and reliability can be improved as well.

### 2.2. Operating Principle

The proposed motor operates on principle of coaxial magnetic gear. According to [4], the pole pairs of flux density space harmonics distribution   *p*_*m*,*k*_  produced by the PM outer-rotor can be deduced as shown in (1), and the speed of the flux density space harmonics   *ω*_*m*,*k*_  is given by (2):

(1) pm,k=|mp2+kNs|,

(2) ωm,k=mp2mp2+kNsω2,

where   *m* = 1,  3,  5,…, *∞*,   *k* = 0,  ±1,  ±2,  ±3,…, ±*∞*,  *p*_2_  is the pole pairs of the PM outer-rotor,   *N*_*s*_  is the number of the magnetic field modulation blocks of stationary ring, and   *ω*_2_  is angular velocity of the PM outer-rotor.

To transmit a steady torque, the pole pairs of stator magnetic field   *p*_1_  must be equal to the pole pairs of the space harmonic flux density distribution   *p*_*m*,*k*_. To utilize the maximum harmonic field,   *m* = 1,   *k* = −1. So,  *p*_1_,  *p*_2_  , and   *N*_*s*_  are governed by

(3) $$\begin{array}{c} p_{1}=N_{s}-p_{2}. \end{array}$$

The speed ratio   *G*_*r*_  is given by

(4) $$\begin{array}{c} G_{r}=\frac{\omega_{1}}{\omega_{2}}=\frac{p_{2}}{p_{2}-N_{s}}=-\frac{p_{2}}{p_{1}}, \end{array}$$

where   *ω*_1_  is the speed of rotating field in stator and the negative sign indicates that the direction of rotating field in stator is opposite to that of the PM outer-rotor.

For the proposed machine,   *p*_1_ = 4, *p*_2_ = 23, *N*_*s*_ = 27, and the speed ratio  *G*_*r*_ = 5.57 : 1.

## 3. Design Criteria

### 3.1. Sizing Equation

To reduce THD of back EMF, fractional slot shorted pitch distribution windings are used in [10, 11]. Assuming the stator winding current is controlled to be sinusoidal and synchronized with the back EMF and leakage inductance and winding resistance are ignored, based on [12], the stator sizing equation of the proposed motor can be expressed as

(5) $$\begin{array}{c} D_{os}^{2}l_{e}=\frac{P_{o}}{\left(\sqrt{2}/4\right)\pi^{2}\eta K_{w}B_{g1}\omega_{2}A_{s}},\; \end{array}$$

where  *D*_*os*_ is outside diameter of stator,  *l*_*e*_  is active axial length,  *P*_*o*_  is output power,  *η* is efficiency,  *K*_*w*_  is armature winding factor,  *B*_*g*1_ is the fundamental amplitude of air-gap flux density,_* *_and  *A*_*s*_  is electric loading.

When  *P*_*o*_ is determined, it can be observed that the main dimensions can be designed based on motor speed and electromagnetic load.

### 3.2. Design Flow

The design flow of the proposed motor is given below.

1. According to the technical requirements, the rated power, speed, torque, and voltage of the motor can be deduced.
2. Based on (5), stator diameter, stator pole pairs, rotor pole pairs, stack length, and stator winding turns can be initialized.
3. Determine the solution domain and the boundary conditions for finite element analysis, generate meshes, and apply the FEM for magnetic field analysis.
4. Evaluate the magnetic field distributions, air-gap flux linkages, and inductances. Hence, the no-load EMF and torque performances can be simulated.
5. Modify the size of motor and winding turns, and repeat steps (3) to (4) until the expected performances can be attained.

## 4. Static Performance Analysis

**Figure 2:**
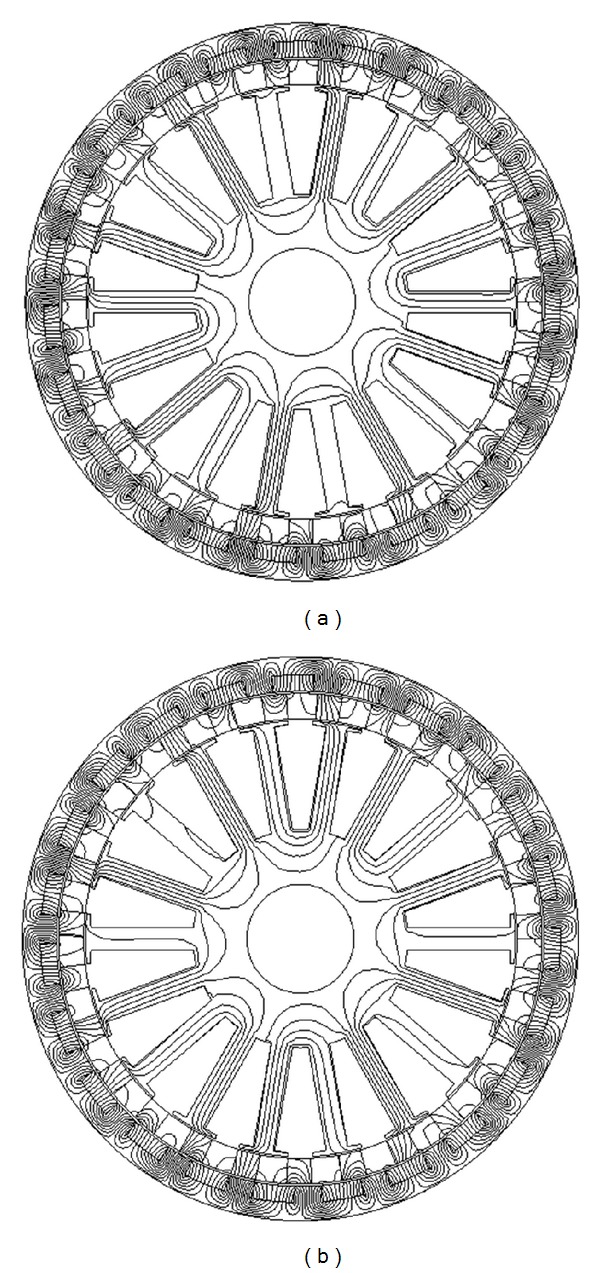
Magnetic field distributions: (a) no-load and (b) full-load.

**Figure 3:**
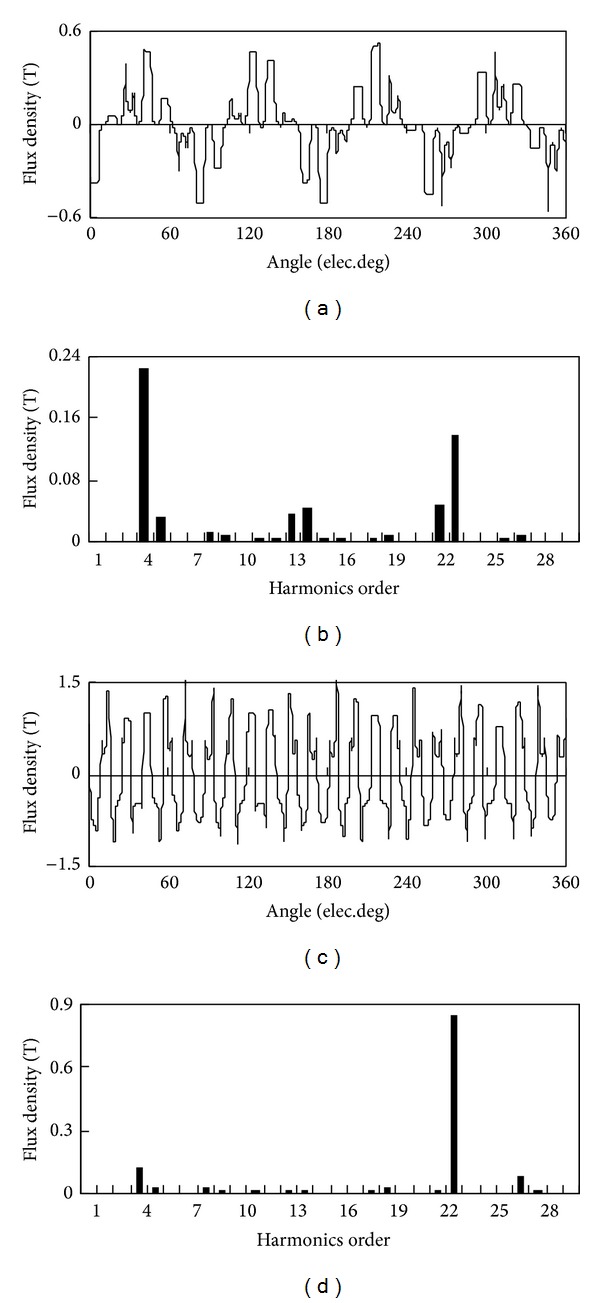
Radial magnetic field waveforms: (a) inner air-gap, (b) inner harmonic spectrum, (c) outer air-gap, and (d) outer harmonic spectrum.

The 2D finite element method (FEA) is employed to analyze the static performance of the proposed motor. The specifications of the proposed motor are listed in Table 1. Figures 2(a) and 2(b) compare the magnetic field distributions at no-load and full-load. It can be observed that most of the flux produced by the PMs embedded in the outer-rotor passes through the modulation blocks of the stationary ring. In Figure 3 are the radial magnetic field waveforms in both the inner air-gap and the outer air-gap at no-load. It can be noted that the largest space harmonic is changed from 23 pole pairs in the outer air-gap to 4 pole pairs in the inner air-gap, which verifies the modulation effect of the stationary ring.

**Figure 4:**
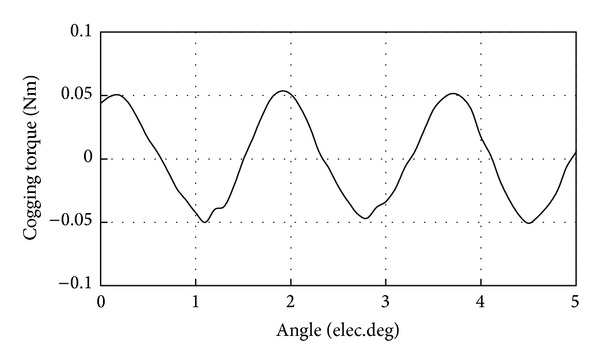
Cogging torque waveform.

Cogging torque is generally a major problem in PM motors [13–16]. In the proposed motor, various techniques such as fractional slot, optimized pole arc width, and optimal number of modulation blocks are adopted to reduce the cogging torque.  Figure 4 shows the cogging torque waveform calculated by the FEA. The magnitude of the cogging torque of the proposed motor is very small, only 0.05 Nm, which is due to the large value of the smallest common multiple between stator pole pairs and the rotor pole pairs.

## 5. Simulation and Experimental Verification

### 5.1. System Configuration and Control Strategy

**Figure 5:**
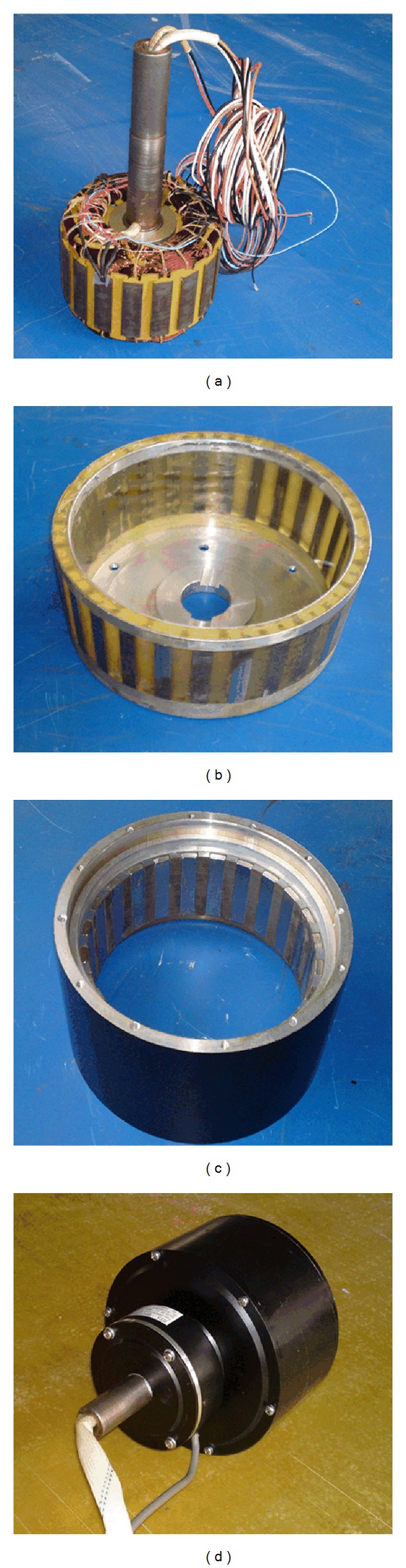
Proposed self-decelerating PM motor: (a) stator, (b) stationary ring, (c) outer-rotor, and (d) assembled prototype.

**Figure 6:**
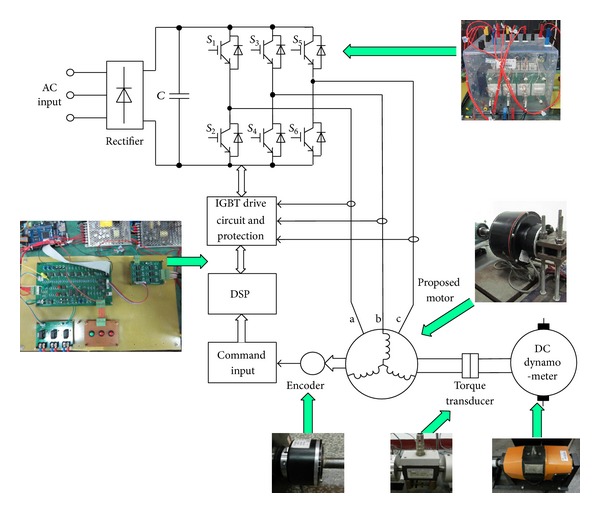
System configuration.

A 500 W target motor is built to verify the validity of the proposed motor as shown in Figure 5. In order to verify the performance of the proposed motor, an integrated power stage is used to drive the prototype, which is composed of a three-phase diode bridge rectifier and an inverter controlled by a DSP. The current inputs, encoder circuit and protection, are also included in the controller circuit board. The system configuration schematic is shown in Figure 6. The drive system includes the prototype motor, a SEMIKRON integrated full-bridge inverter, and a DSP-based controller. An incremental encoder with 1024 pulses/revolution is employed in this set-up. A DC dynamometer is a variable load. The output torque of the proposed machine is measured by a HBM torque transducer, and all data are acquired via a computer.

**Figure 7:**
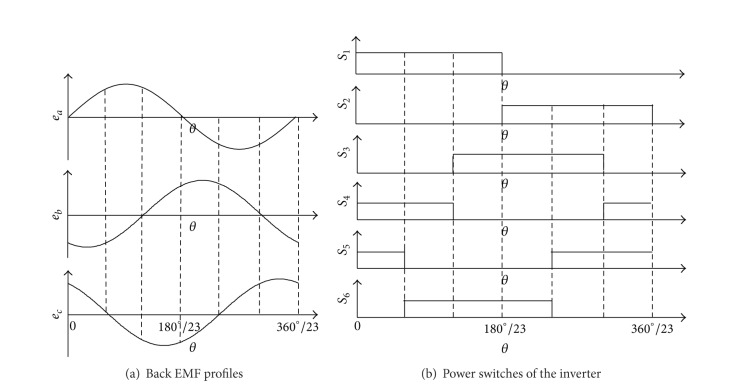
Control logic.

Because the back EMFs are sinusoidal, the turn-on angle can be selected to ensure that phase current has fixed phase difference with the back EMF.  Based on the back EMF profiles (*e*_*a*_, *e*_*b*_, *e*_*c*_) shown in Figure 7(a), the control logic for all power switches of the inverter (*S*_1_, *S*_2_, *S*_3_, *S*_4_, *S*_5_, *S*_6_) can be determined as shown in Figure 7(b).

### 5.2. Simulation and Experimental Results

**Figure 8:**
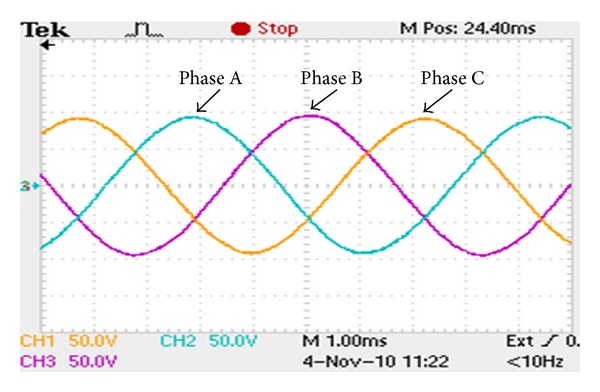
Measured three-phase output voltages at no-load.

**Figure 9:**
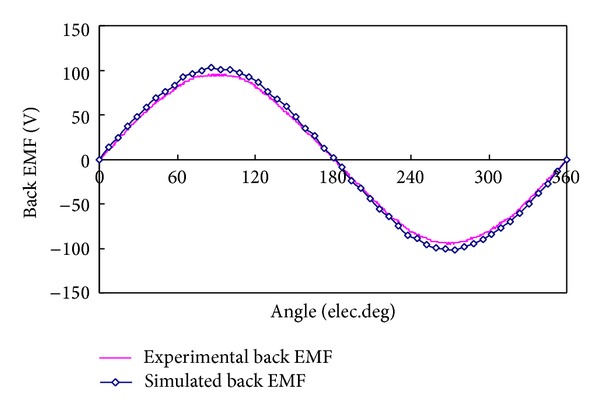
Comparison of back EMF variations between FEA and experimental results.

**Figure 10:**
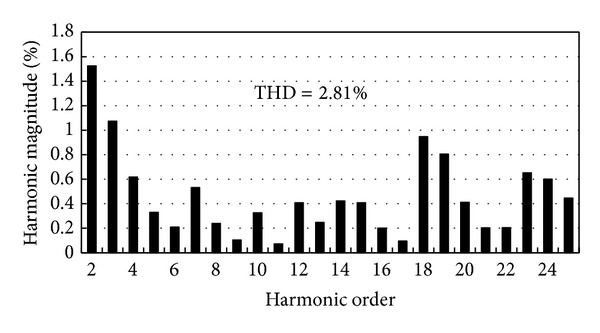
Experimental back EMF harmonic analysis of phase A.

Figure 8 shows the measured open-circuit phase-to-neutral back EMF waveform at the rated speed of 400 rpm.  Figure 9 shows the measured back EMF waveform of phase A for the prototype together with the predicted waveform obtained by FEA. It can be seen that the measured back EMF waveform well agrees with the simulation one and both are very sinusoidal. The harmonic analysis of the back EMF of phase A deduces that the THD is 2.81%. The corresponding histogram is depicted in Figure 10. Also, from Figure 8, it can be observed that the experimental back EMF frequency is 153 Hz. It indicates that the rotational speed of the magnetic field in the inner air-gap is 2299 rpm (namely, 5.749 times that of outer-rotor speed) which agrees with theoretical value of 5.75. The result confirms the validity of the design. Meanwhile, there is a slight difference between the amplitudes of these two waveforms. The FEA-predicted back EMF waveform for the proposed motor has the amplitude 5.88% higher than that of the measured value. This difference is caused by two reasons. Firstly, there are inevitable errors caused by manufacturing process. Secondly, the 2-D FEA is definitely less accurate than the 3-D FEA.

**Figure 11:**
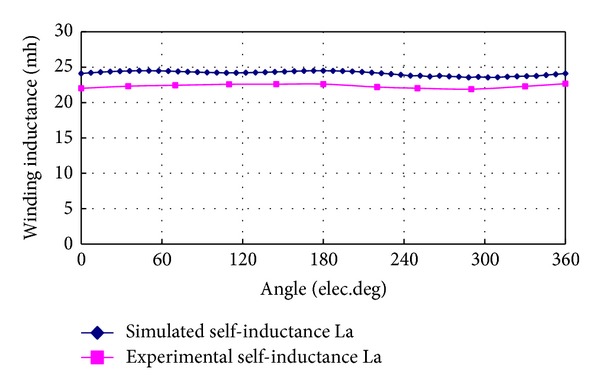
Comparison of self-inductances between FEA and experimental results.

Figure 11 shows the comparison of self-inductance of phase A obtained from experiment and FEA simulation. The experimental result agrees with the simulation one very well, and the maximum error is about 8.2% which is mainly due to the manufacturing imperfection and end effect. It also illustrates that the self-inductance of phase A is approximately constant, so the rotor magnetic circuit is almost symmetric.

**Figure 12:**
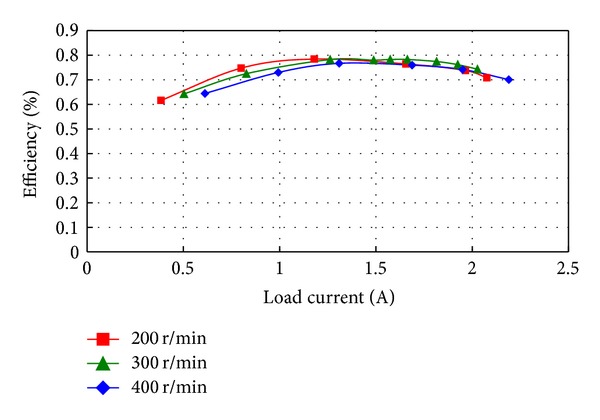
Measured efficiency versus load current at different speeds.

The efficiency of the proposed motor is measured at different load currents and outer-rotor speeds. As shown in Figure 12, the measured efficiency is higher than 75% over a wide load range at different speeds, whereas the maximum efficiency is about 80% which occurs at 200 rpm. The power losses of motor include copper losses, hysteresis losses of the core, eddy current losses of the PM, mechanical losses, and stray losses. Assuming the armature current is sinusoidal, it has the same frequency and synchronizes with the back EMF. A 2-D FEA model is built to analyze the losses of the motor at 400 rpm and full-load. It turns out that the copper losses are 13.32 W. The hysteresis losses of the stator core, rotor core, and modulation blocks in the stationary ring are 2.55 W, 0.72 W, and 1.38 W, respectively. And the eddy current losses of them are 3.66 W, 1.61 W, and 2.46 W, respectively. All the iron losses count up to 12.38 W. The eddy current losses of the PM are 1.6 W. The stray losses and mechanical losses are totally estimated to 10 W, about 2% of the rated power. The simulated losses are less than the experimental ones, mainly because of the neglected end effect, the harmonics of the input current introduced by the power devices in the experiment, the inaccuracy of the iron losses calculation method for not taking the influence of DC-biased magnetic induction into consideration, and the inevitable manufacturing imperfection. It should be noted that the system efficiency of the FMDD motor is relatively lower than a standard direct drive PM machine, because the operation principle of the motor is based on utilizing the harmonic field, which will introduce large amounts of iron losses. Nevertheless, such reduction of efficiency is well outweighed by the uniqueness of self-deceleration with no gear.

**Figure 13:**
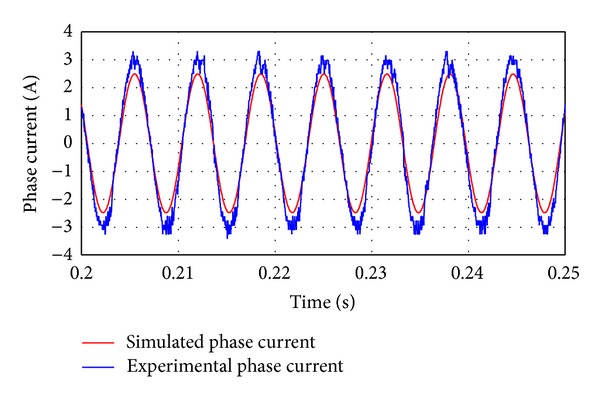
Comparison of phase current between simulation and experimental results.

**Figure 14:**
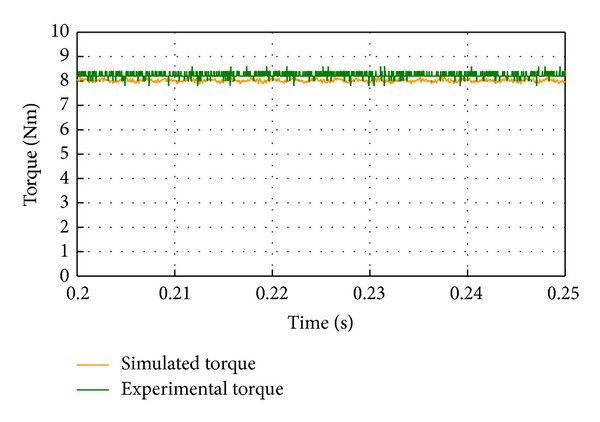
Comparison of output torque between simulation and experimental results.

To assess the performance of the proposed motor, the hysteresis current control with *I*_*d*_ = 0 vector control method is implemented.  Figure 13 shows the comparison of phase currents between the simulation and experimental results under the rated load of 8 Nm at 400 rpm. It should be noted that phase current is effectively controlled to be sinusoidal and the two results agree well. Nevertheless, the amplitude of the experimental result is slightly higher than the simulation one. It is expected since the simulation result has not taken into account the parasitic losses which require additional current for the desired performance. The output torque waveforms of the experimental and the simulation results are shown in Figure 14. It can be observed that the torque ripple is very low which is due to the fact that phase current is synchronized with the back EMF when the *I*_*d*_ = 0 control is used, and there is a large value of the smallest common multiple between stator slot number and the rotor pole pairs. The slight difference between them is mainly caused by the discrepancy of damping torque in the simulation and the experiment. Moreover, the measured output torque of 8.2 Nm validates the design of modulation block ratio for the stationary ring. The measured torque density is about 9 kNm/m^3^; it is lower than a standard direct drive PM machine; this is primarily due to the manufacturing imperfection and no optimization of the machine design. The maximization of the torque density will be addressed in the future work by employing concentrated, single tooth windings and fractional numbers of slots/pole/phase, with high slot fill factors and short end-windings.

**Figure 15:**
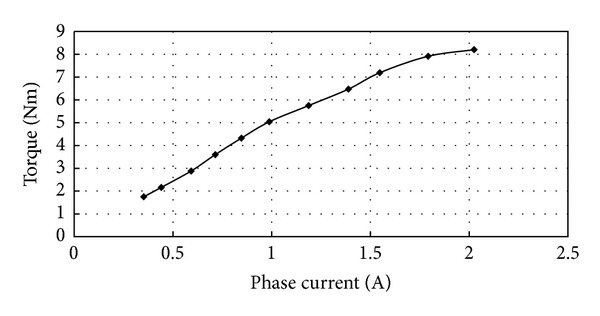
Measured torque versus current at the speed of 400 rpm.

**Figure 16:**
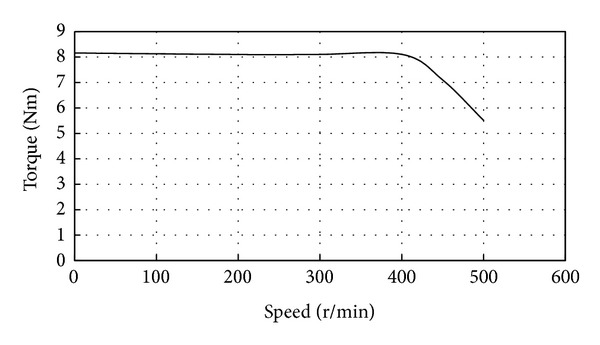
Measured torque-speed characteristic.

Finally, the torque-current and torque-speed characteristics are measured as shown in Figures 15 and 16, respectively.  Figure 15 shows the torque versus current at the speed of 400 rpm; it shows a linear torque and current relationship.  Figure 16 verifies that the proposed magnetic-geared PM motor can guarantee a constant torque over a wide speed range. Hence, this FMDD motor can operate in a low speed and provide a steady torque output without using a mechanical gearbox.

## 6. Conclusion

This paper has designed, analyzed, and implemented a flux-modulated direct drive motor with two air-gaps using the sizing equation and FEA. The configuration and operating principle of the motor are described. Field distribution, inductance characteristics, and cogging torque of the motor are investigated using the 2-D FEA. The evaluation of system performances is conducted by computer simulation. To verify the performance of the proposed motor, an experimental system is built. The back EMF, efficiency, torque-speed characteristic, and torque output have been measured, and good agreement between the simulation and experimental results has been found. Both the simulation and experimental results confirm that thisflux-modulated direct drive motor can achieve speed changing without the gearbox and provide a steady output torque with a simple control method.

## Figures and Tables

**Table 1:** Specifications of proposed PM motor.

Rated power (W)	500
Rated phase voltage (V)	75
Rated speed of outer-rotor (rpm)	400
Rated frequency (Hz)	153
Outer-rotor pole-pair number	23
Stationary ring modulation block number	27
Stator slot number	18
Outside diameter of outer-rotor (mm)	155.2
Inside diameter of outer-rotor (mm)	136.2
Outside diameter of stator (mm)	120
Air-gap length (mm)	0.6
Active axial length (mm)	48
Voltage constants *K*_*e*_ (volts/1000 rpm)	480.7
Torque constants *K*_*t*_ (oz-in/A)	650
